# Local delivery of HMGB1 in gelatin sponge scaffolds combined with mesenchymal stem cell sheets to accelerate fracture healing

**DOI:** 10.18632/oncotarget.16887

**Published:** 2017-04-06

**Authors:** Deting Xue, Wei Zhang, Erman Chen, Xiang Gao, Ling Liu, Chenyi Ye, Yanbin Tan, Zhijun Pan, Hang Li

**Affiliations:** ^1^ Department of Orthopaedics, 2nd Affiliated Hospital, School of Medicine, Zhejiang University, Hangzhou 310009, P.R. China; ^2^ Department of Nephrology, Hangzhou Hospital of Traditional Chinese Medicine, Hangzhou 310007, P.R. China

**Keywords:** cell sheets, HMGB1, fracture healing, gelatin sponge scaffold, tissue engineering

## Abstract

Fracture nonunion and delayed union continue to pose challenges for orthopedic surgeons. In the present study, we combined HMGB1 gelatin sponges with MSC sheets to promote bone healing after surgical treatment of rat tibial fractures. The HMGB1 gelatin sponge scaffolds supported the expansion of mesenchymal stem cells (MSCs) and promoted the osteogenic differentiation of MSCs and MSC sheets. Lentiviral vectors were then used to overexpress HMGB1 in MSCs. The results indicated that HMGB1 promotes the osteogenic differentiation of MSCs through the STAT3 pathway. Both siRNA and a STAT3 inhibitor downregulated STAT3, further confirming that HMGB1 induces the osteogenic differentiation of MSCs partly via the STAT3 signal pathway. In a rat tibial osteotomy model, we demonstrated the ability of HMGB1 gelatin sponge scaffolds to increase bone formation. The addition of MSC sheets further enhanced fracture healing. These findings support the use of HMGB1-loaded gelatin sponge scaffolds combined with MSC sheets to enhance fracture healing after surgical intervention.

## INTRODUCTION

Fracture nonunion or delayed union continues to pose challenges for orthopedic surgeons. The overall rate of delayed union or nonunion is 5–10%, although nonunion rates differ for different types of fractures, ranging from up to 18.5% in the tibial diaphysis to 1.7% in the femoral shaft after reamed nailing [1–3]. In recent years, tissue engineering has shown promise in bone regeneration.

Growth factors play key roles in the “diamond concept” of fracture healing [4, 5]. Combinations of exogenous bone morphogenetic proteins (BMPs) and scaffolds in animal models of tissue engineering improve healing [6, 7]. However, clinically, BMPs have failed to exhibit the anticipated efficacies, both during fracture healing and when used to treat tibial non-union [8–10]. Thus, other effective osteogenic growth factors are required. HMGB1 is one of the principal local stressors of the family of molecules termed the danger-associated molecular patterns (DAMPs). Recently, HMGB1 has been shown to promote osteogenesis. Hsu et al. [11] reported that HMGB1 promoted the osteogenesis of adipose tissue-derived stem cells. We earlier showed that HMGB1 stimulated MSC osteogenesis and migration [12, 13]. Thus, the potential of HMGB1 as an osteogenic cytokine augmenting local bone regeneration merited further investigation.

Osteogenic cells, scaffolds, growth factors, and the mechanical environment are four important elements in bone regeneration [14]. MSCs are potential to differentiate into a variety of cell types, including adipocytes, chondrocytes, and osteoblasts. Accordingly, their use in osteoblast differentiation and bone regeneration has been proposed [15, 16]. However, in tissue engineering studies, the number of implanted stem cells is limited by the low surface-to-volume ratio of the scaffolds [17]. A possible solution to this problem is the use of stem cell sheets [18] as a source of high-density cells. In addition, cell sheets can be easily detached from the culture substrate without proteolytic treatment, thus leaving intact critical cell-surface proteins, such as ion channels, growth factor receptors, and cell-to-cell junction proteins [18]. The homotypic layering of cell sheets may act as an artificial periosteum in bone regeneration [19, 20].

Scaffolds are another key element in bone regeneration. Gelatin sponges is a natural material and it offers a porous structure, and excellent biocompatibility, while their hydrophilicity recommends them as effective carriers for the local delivery of HMGB1.

In the present work, we try to combine HMGB1 gelatin sponges with MSC sheets and access its role on the bone healing in a rat tibial osteotomy model. Our results suggest that local delivery of HMGB1 by gelatin sponges enhances bone formation during tissue engineering.

## RESULTS

### Scaffold characterization

**Figure 1:**
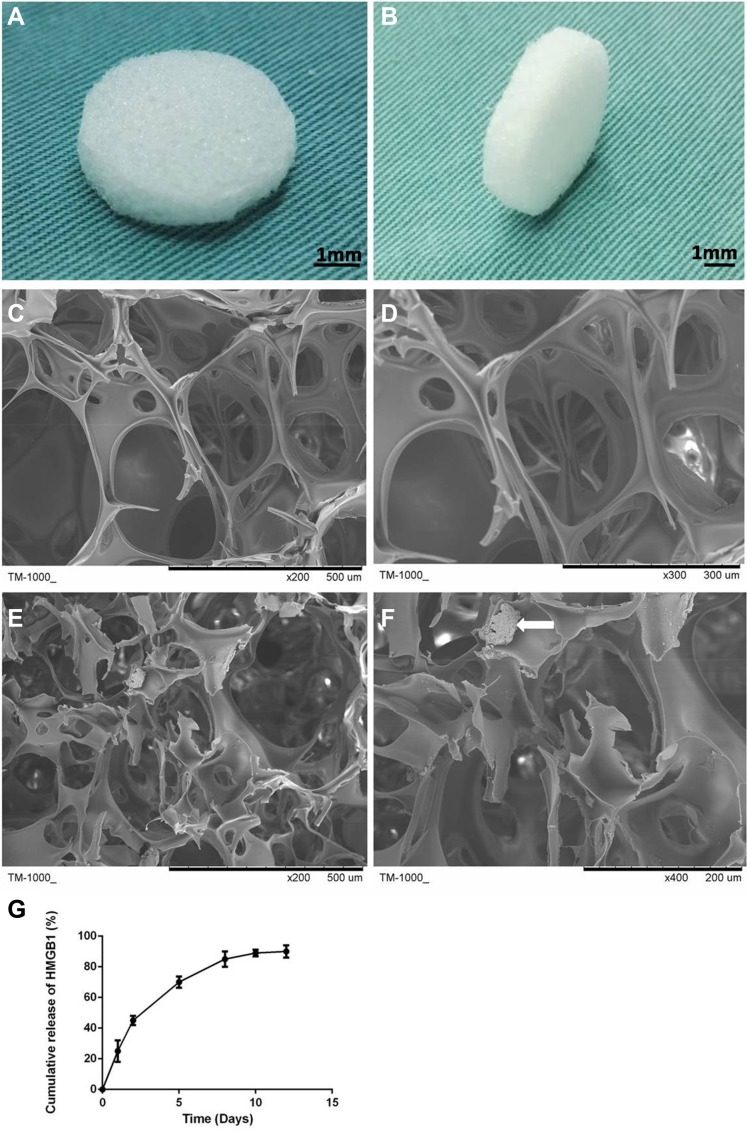
Disc-shaped gelatin sponge scaffolds (**A**, **B**). Scanning electron microscopy (SEM) micrograph of the gelatin sponge and gelatin-HMGB1 scaffolds. (**C**) The micropore structure and (**D**) the pore wall surface of the gelatin scaffold. (**E**) The micropore structure and (**F**) the pore wall surface of the gelatin-HMGB1 scaffold. Crystallized HMGB1 protein is distributed in the pore wall. The white arrow points to the crystalline aggregations of HMGB1 protein. (**G**) Cumulative release of HMGB1 from a gelatin sponge scaffolds over 12 days.

The gelatin sponge was cut into discs (Figure 1) and imaged using SEM. The resulting images showed the homogeneously interconnected and open pore structure of both the gelatin sponge and gelatin-HMGB1 scaffolds (Figure 1). Crystallized HMGB1 protein was distributed on the pore walls of the gelatin-HMGB1 scaffolds (Figure 1). The porosity of the gelatin sponge and gelatin-HMGB1 scaffolds was 78 ± 1.1% and 76.3 ± 1.8%, respectively.

### *In vitro* HMGB1 release kinetics

The cumulative release of HMGB1 from the gelatin scaffolds was measured and plotted (Figure 1G). The resulting release curve exhibited a sharp initial burst on day 1, at which time ~25% of the total amount of HMGB1 had been released from the scaffold. This abrupt release may have been due to the free protein remaining at the pore surface that was not conjugated efficiently by the scaffold. Stable release was maintained such that ~85% of the loaded HMGB1 had been released by day 7.

### The characteristics of MSC and the MSC sheets

**Figure 2:**
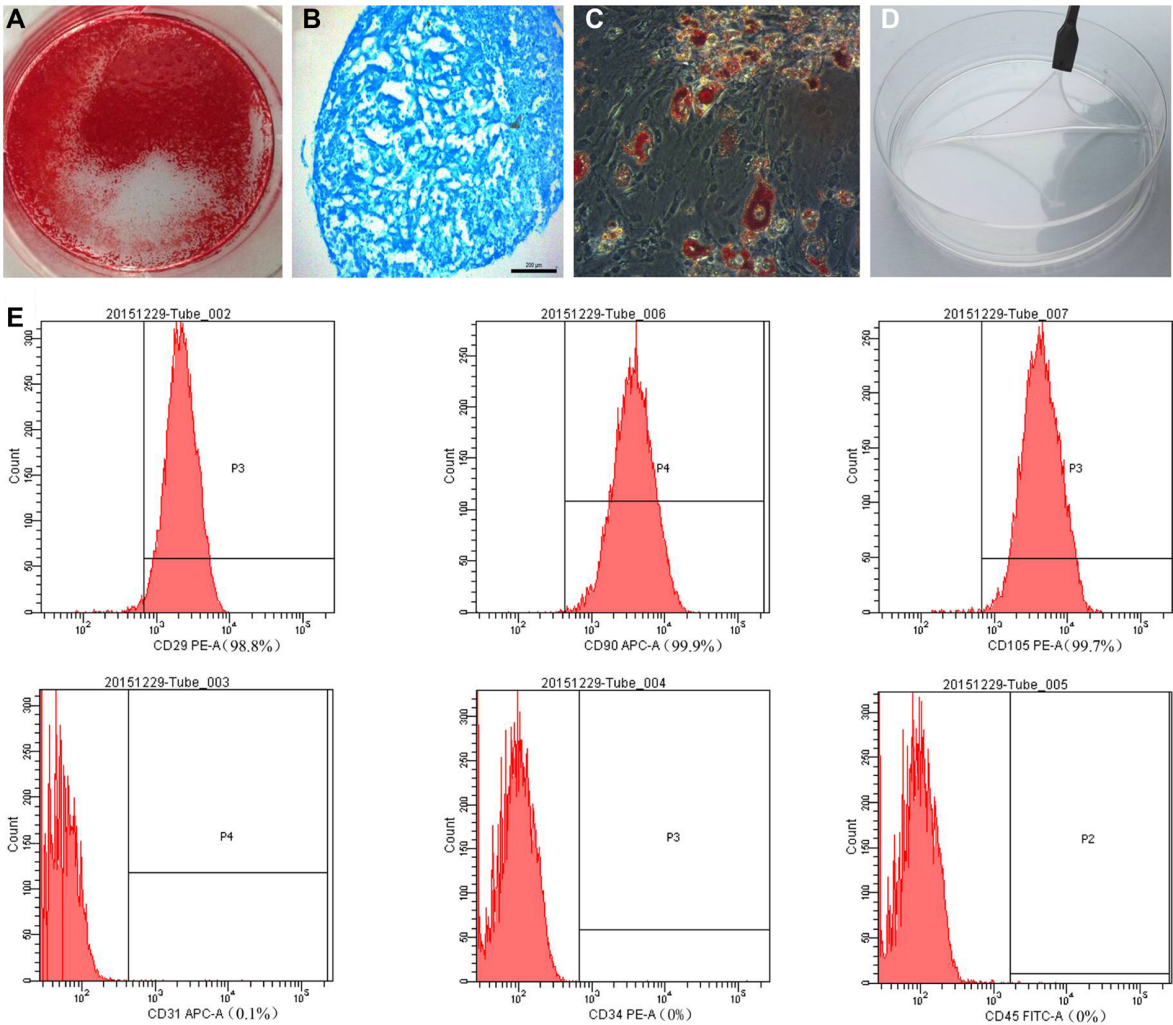
(**A**) Alizarin red, (**B**) Alcian blue, and (**C**) oil red O staining of mesenchymal stem cells (MSCs) after osteogenic, chondrogenic (pellet culture), and adipogenic induction, respectively. (**D**) The MSC sheet was easily detached from the dish. (**E**) MSC-specific marker expression was assessed by flow cytometry.

The morphology of the isolated MSCs was monitored and the multipotency of the isolated cells was examined by inducing them to differentiate toward osteoblasts, chondrocytes, and adipocytes. Osteogenic induction was confirmed by ARS, in which red staining represented mineralization (Figure 2A). Abundant GAG deposition, characteristic of chondrocytes, was confirmed by the extensive blue staining with Alcian blue (Figure 2B), while adipogenic induction was demonstrated by the formation of red oil droplets following oil red O staining (Figure 2C). We used flow cytometry to assess the expression levels of MSC-specific markers. The isolated MSCs were strongly positive for CD29, CD90, and CD105, and consistently negative for CD31, CD34, and CD45 (Figure 2E).

To obtain MSC sheets, MSCs seeded on a culture dish were allowed to proliferate for 14 days, after which the newly formed sheets were easily detached from the dish using a scraper (Figure 2D). The viabilities of cell sheets and MSCs at 90% confluence were 82.5 ± 1.85% and 91.75 ± 0.85% (*P <* 0.01), respectively (Supplementary Figure 1). The differentiation assay showed that the cell sheet continued to exhibit high-level multipotency (Supplementary Figure 2).

### The effect of HMGB1 and HMGB1-gelatin sponge scaffold on MSC expansion

**Figure 3:**
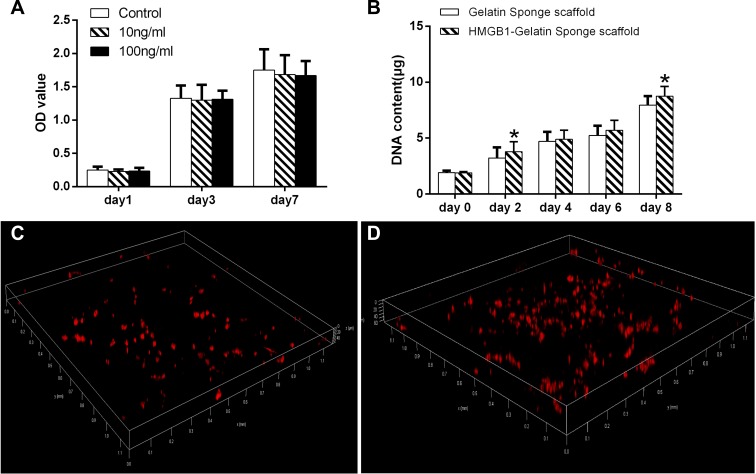
(**A**) The effect of different concentrations of HMGB1 on MSC expansion. (**B**) The DNA content of MSCs in the gelatin sponge and HMGB1-gelatin sponge scaffolds after 0, 2, 4, 6 and 8 days of culture. Each scaffold was seeded with 100 μL of cells to achieve a density of 1 × 10^6^ cells/scaffold. *Significant (*P* < 0.05) difference between the HMGB1-gelatin and gelatin sponge scaffolds at the same time point. (**C**) Confocal images of the distribution of CM-DiI-stained MSCs in (C) the gelatin sponge and (**D**) the HMGB1-gelatin sponge scaffolds obtained after 7 days of *in vitro* culture. Three-dimensionally rendered z-stack images were evaluated.

The addition of 100 ng HMGB1/mL to the culture medium resulted in mild, but not significant (compared to the untreated control group), inhibition of MSC expansion (Figure 3A).

The proliferation of MSCs in the scaffolds was assessed in a DNA assay using Hoechst33258 dye. By day 8, the DNA content of cells entrapped in the gelatin sponge scaffolds and HMGB1-gelatin sponge scaffolds had increased 4.2- and 4.6-fold, respectively, compared with day 0. On day 8, the DNA content of the cells in the HMGB1-gelatin sponge scaffold was significantly higher than that of cells in the gelatin sponge scaffolds (both *P <* 0.05), indicating that HMGB1 could improve gelatin sponge scaffold biocompatibility to support the proliferation of MSCs *in vitro* (Figure 3B). However, in the corresponding plate cultures, the number of MSCs in the HMGB1-treated and control groups did not significantly differ (Figure 3A).

To evaluate the distribution of the cells in the scaffolds, MSCs were labeled with CM-DiI fluorescence and imaged by CLSM. The labeled cells showed uniformly red fluorescence. In the three-dimensional rendering of the z-stack images, by day 9 CM-DiI-MSCs were more abundant in the HMGB1-gelatin sponge scaffolds than in the gelatin sponge scaffolds (Figure 3C, 3D).

### The effect of HMGB1 on the osteogenetic differentiation of MSCs and MSC sheets

**Figure 4:**
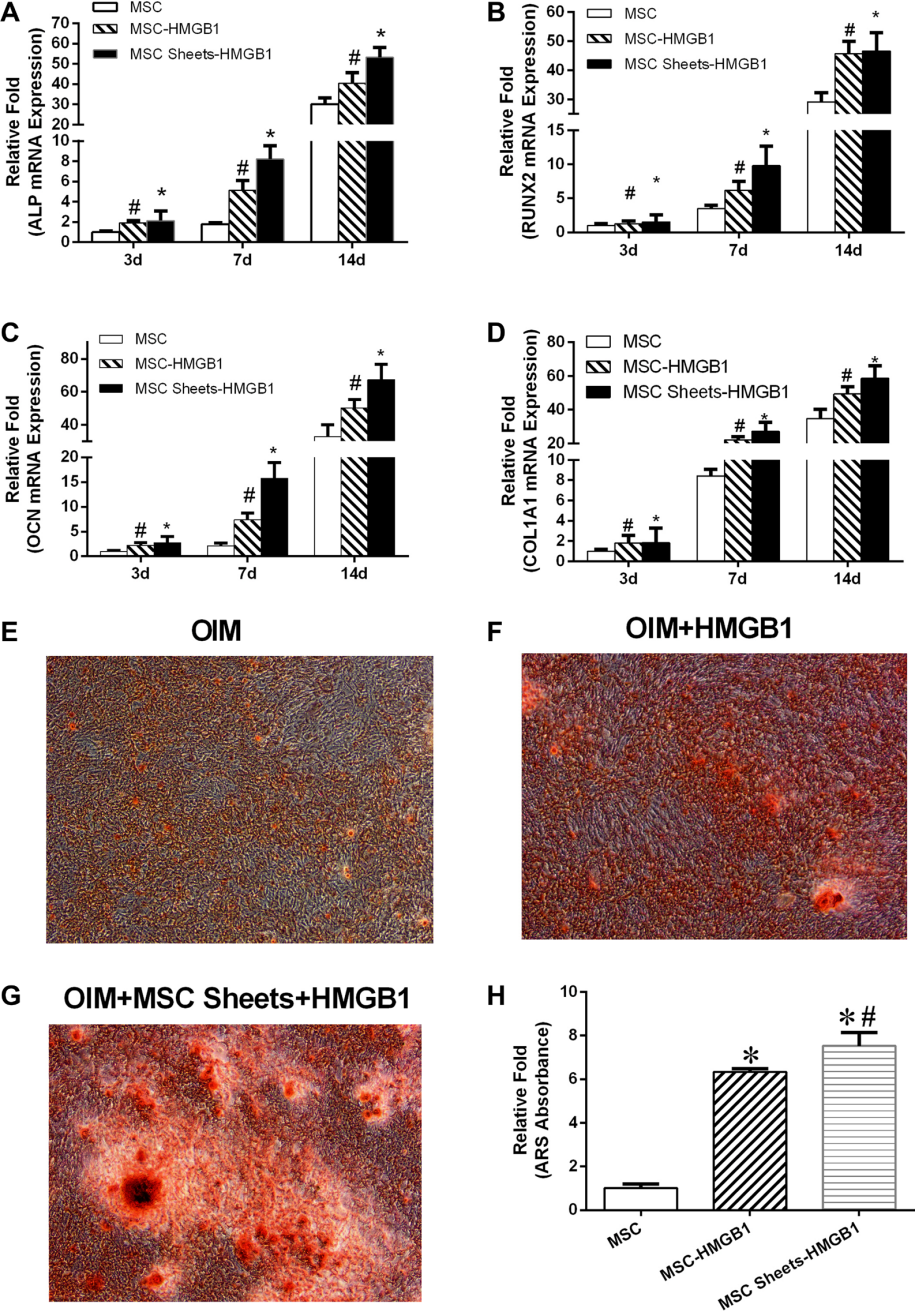
The levels of alkaline phosphatase (ALP), BMP2, OCN and OPG gene expression in MSC, MSC-HMGB1, and MSC sheets-HMGB1 groups at days 3, 7, and 14 (**A**–**D**). The cells were stained with alizarin red S (ARS) on day 28 (**E**–**G**). Mineralization was quantified by the extraction of ARS-stained cells using 10% cetyl pyridinium chloride (CPC). (**H**) *indicates *P <* 0.05 vs. the MSC group and ^#^indicates *P <* 0.05 vs. the MSC-HMGB1 group.

The effect of HMGB1 on the osteogenetic differentiation of MSCs and MSC sheets was assessed using RT-PCR. The results showed the significantly higher expression of ALP, RUNX2, OCN, and COL1A1 in both groups than in the MSC group. In addition, the upregulation of ALP, RUNX2, OCN and COL1A1 was significantly higher in the MSC sheet-HMGB1 group than in the MSC-HMGB1 group, both at day 7 and day 14 (Figure 4A–4D).

Calcification was accelerated in cells cultured for 28 days in the presence of 100 ng HMGB1/mL rather than in OIM alone, and was significantly greater in HMGB1-treated MSC sheets than in similarly treated MSCs (Figure 4E–4G). Mineralization, quantified by the extraction of ARS-stained cells with 10% cetyl pyridinium chloride (CPC), was significantly higher in the MSC-HMGB1 and MSC sheet-HMGB1 groups than in the MSC group (Figure 4H).

### A role for STAT3 in HMGB1-induced osteogenetic differentiation and mineralization by MSCs

**Figure 5:**
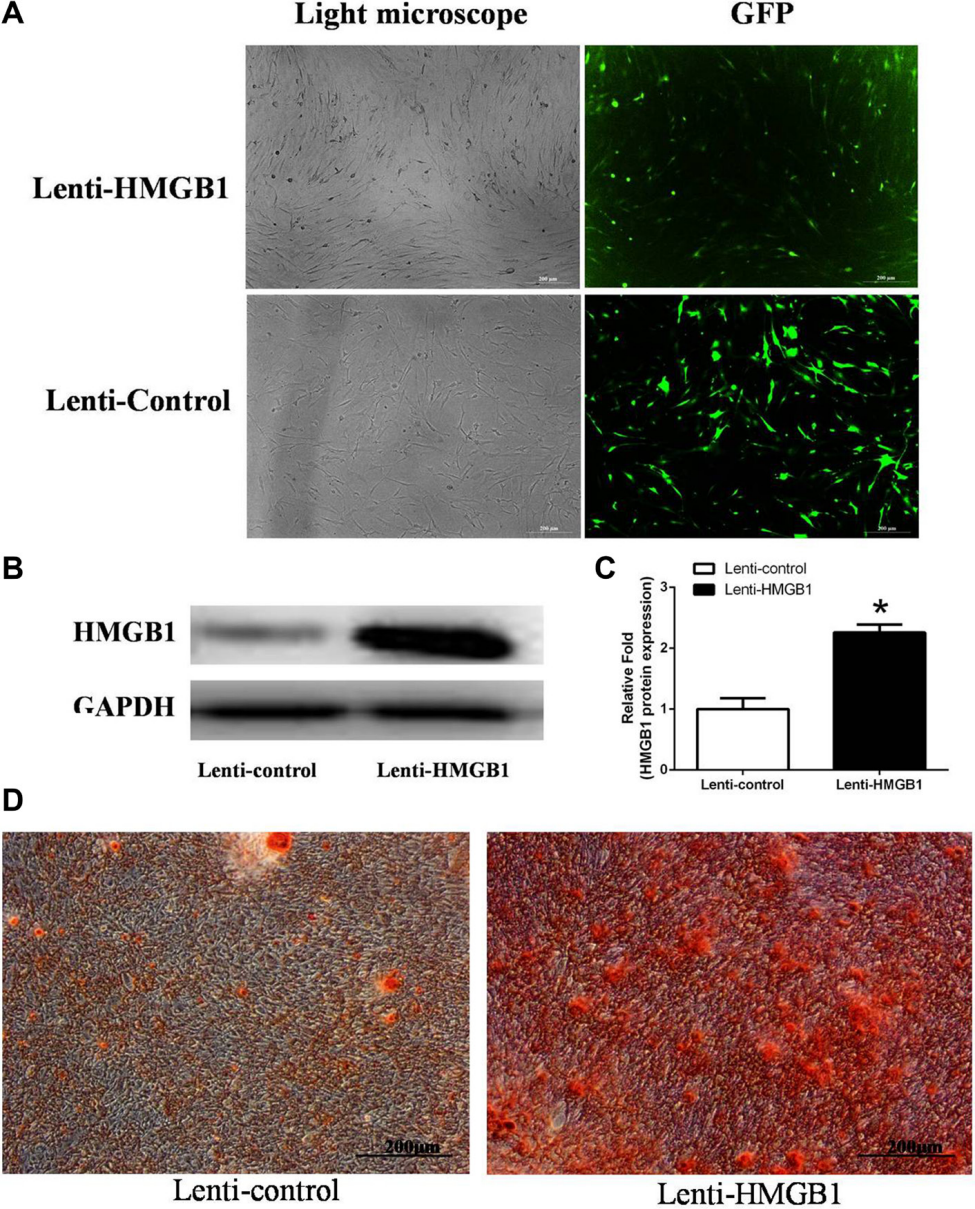
(**A**) MSCs transfected with lentivirus to overexpress HMGB1 or the control green fluorescent protein (GFP) gene. (**B**, **C**) Levels of HMGB1 protein expression were determined by western blot analysis in the Lenti-HMGB1 and Lenti-control groups. (**D**) ARS staining was performed on day 21. *indicates *P <* 0.05 vs. the Lenti-control group.

The signal pathways involved in the accelerated calcification by MSCs in response to HMGB1 were investigated using a lentiviral vector system to efficiently overexpress HMGB1 in these cells. HMGB1 overexpression, quantified based on the ratio of GFP-positive cells to the total number of third-generation MSCs, was detected in > 70% of the total cells (Figure 5A). The western blot result showed that HMGB1 was overexpressed in the transfected cells by 2.26-fold compared to the control group (Figure 5B, 5C). After 21 days of culture in OIM, ARS staining revealed more calcification deposits in the lenti-HMGB1 group than in the lenti-control group (Figure 5D).

**Figure 6:**
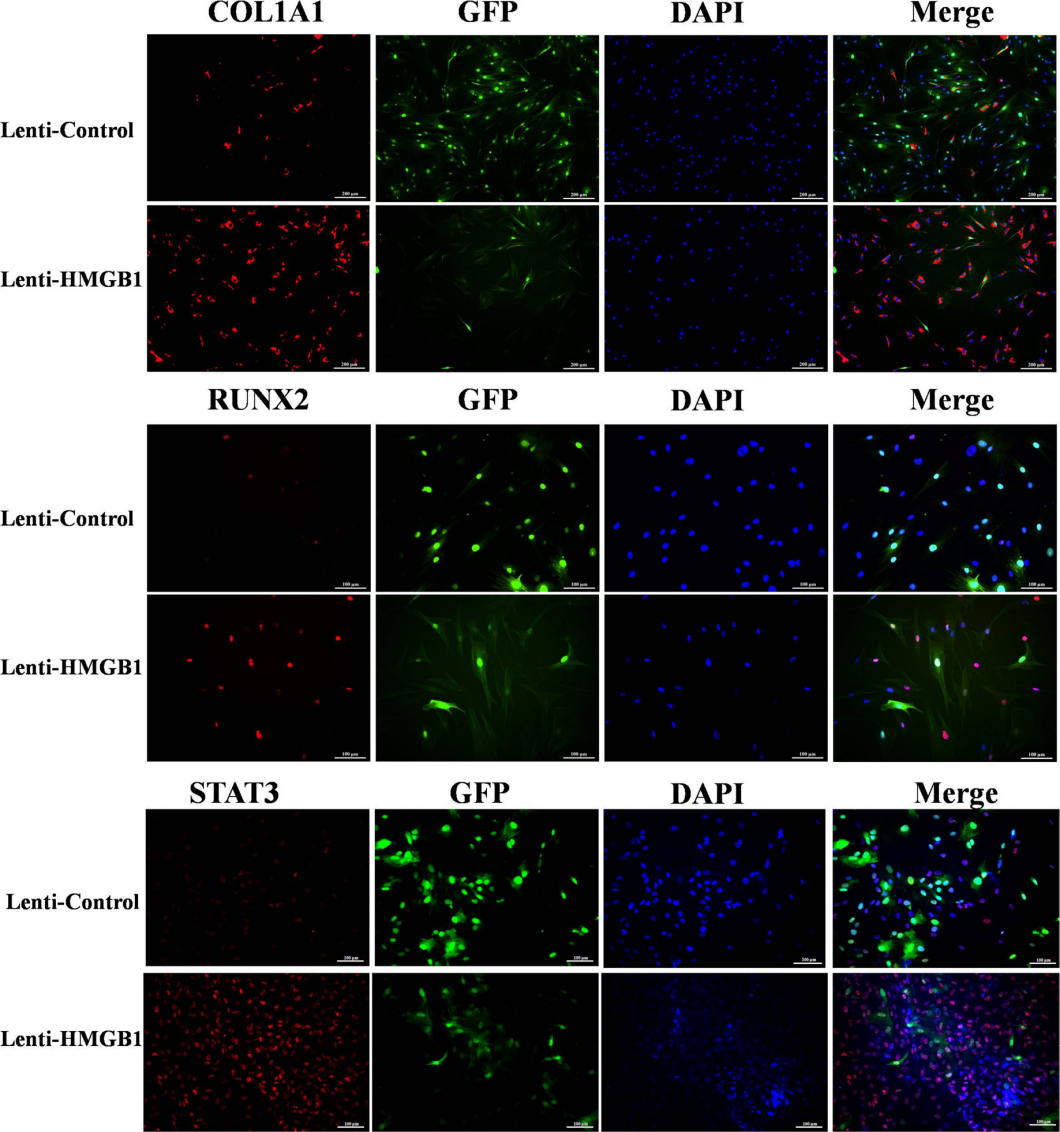
The immunofluorescence of collagen type I, runt-related transcription factor 2 (RUNX2), and phosphorylated STAT3 (p-STAT3) in Lenti-HMGB1 MSCs and lenti-control MSCs after 24 h of culture in osteogenic induction medium (OIM)

A potential role for STAT3 in the HMGB1 signaling pathway during osteogenesis was investigated using immunofluorescence. The results showed an increase in RUNX2 and COL1A1 protein expression in HMGB1-overexpressing cells 24 h after osteogenetic induction (Figure 6). There was little expression of phosphorylated STAT3 (p-STAT3) in the control cells of the OIM cultures. However, after 24 h of culture in OIM, the levels of p-STAT3 were significantly higher in the lenti-HMGB1 MSC group than in the control group (Figure 6).

**Figure 7:**
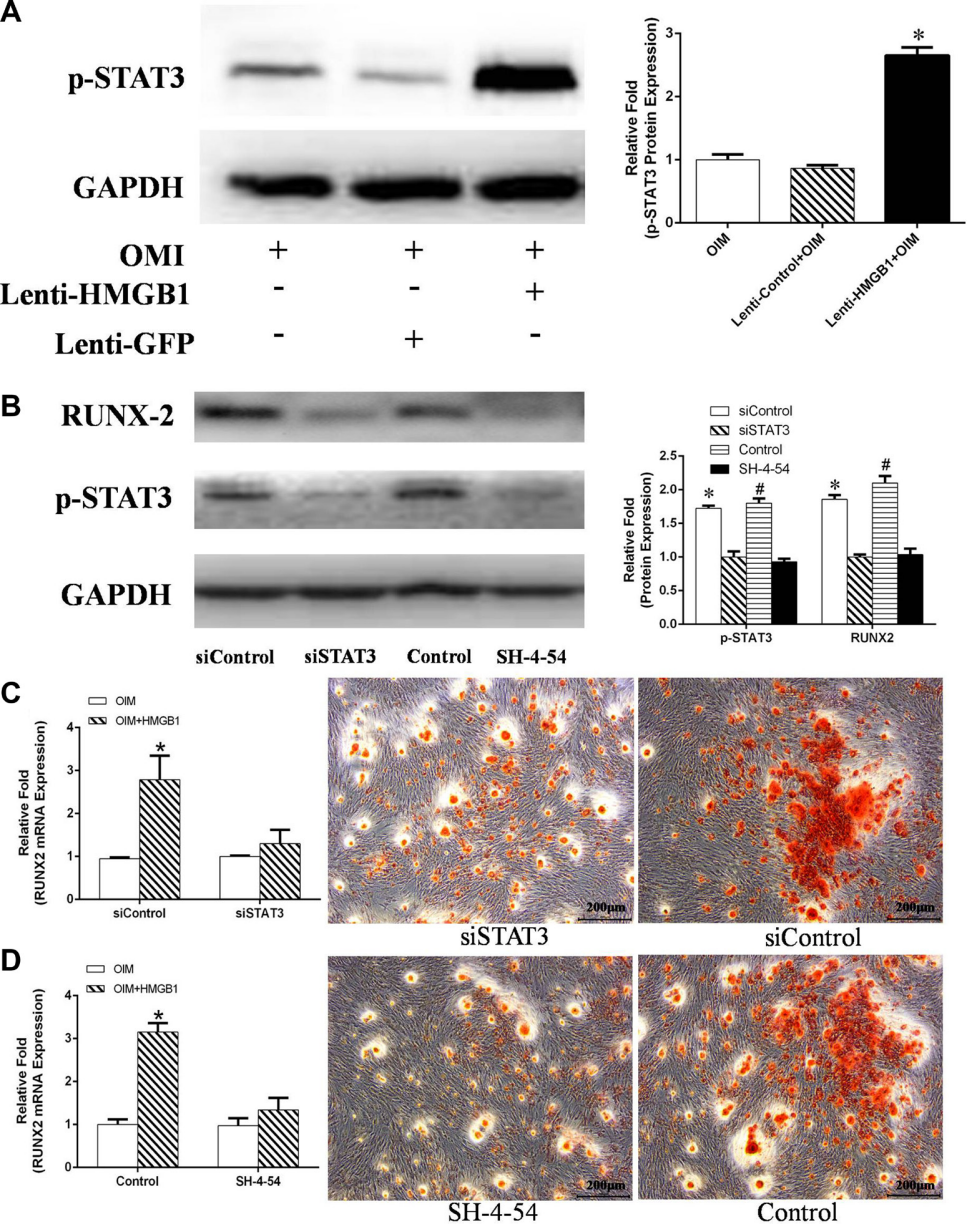
(**A**) MSCs were transfected with Lenti-HMGB1 or, as a control, Lenti-control GFP and then cultured in OIM for 24 h, after which the expression of p-STAT3 was detected by western blotting. Protein expression levels were normalized to GAPDH. *P < 0.05 vs. the OIM group. (**B**) MSCs were transfected with siSTAT3 or a control siRNA (siControl) for 2 days, or pretreated with the STAT3 inhibitor SH-4-54 for 30 min; non-inhibitor-treated MSCs served as a control. The MSCs were then stimulated with HMGB1 for 3 days. The expression of p-STAT3 and RUNX-2 was detected by western blotting and the expression levels were normalized to GAPDH. *P < 0.05 vs. the siControl group, ^#^*P <* 0.05 vs. the control group. (**C**, **D**) Transfected MSCs were cultured in OIM with or without HMGB1 (100 ng/mL) and then stained with ARS on day 21. Total RNA was isolated from siSTAT-transfected MSCs or SH-4-54-pretreated MSCs cultured for 24 h in OIM in the presence of HMGB1. RUNX2 mRNA expression determined by real-time PCR. *P < 0.05 vs. the OIM group.

Further evidence that p-STAT3 is expressed by lenti-HMGB1 MSCs during their culture in OIM was obtained by western blotting, which showed marked upregulation of p-STAT3 after 24 h (Figure 7A). The effect of STAT3 inhibition on the HMGB1-induced osteogenic differentiation of MSCs was determined by culturing the cells in OIM for 3 days, after their transduction with STAT3 siRNA or after the addition of the STAT3 inhibitor SH-4-54. Protein expression levels were again assessed by western blotting. STAT3 protein levels were effectively suppressed by either siRNA or the STAT3 inhibitor SH-4-54. In addition, the suppression of STAT3 resulted in significant inhibition of RUNX-2 protein levels in OIM-cultured cells (Figure 7B), both in siSTAT3-treated and SH-4-54-treated cells (Figure 7C, 7D). Moreover, cells treated with either inhibitor contained fewer matrix mineralizaton deposits at day 21 of osteogenic differentiation (Figure 7C, 7D).

### Radiographic examination

**Figure 8:**
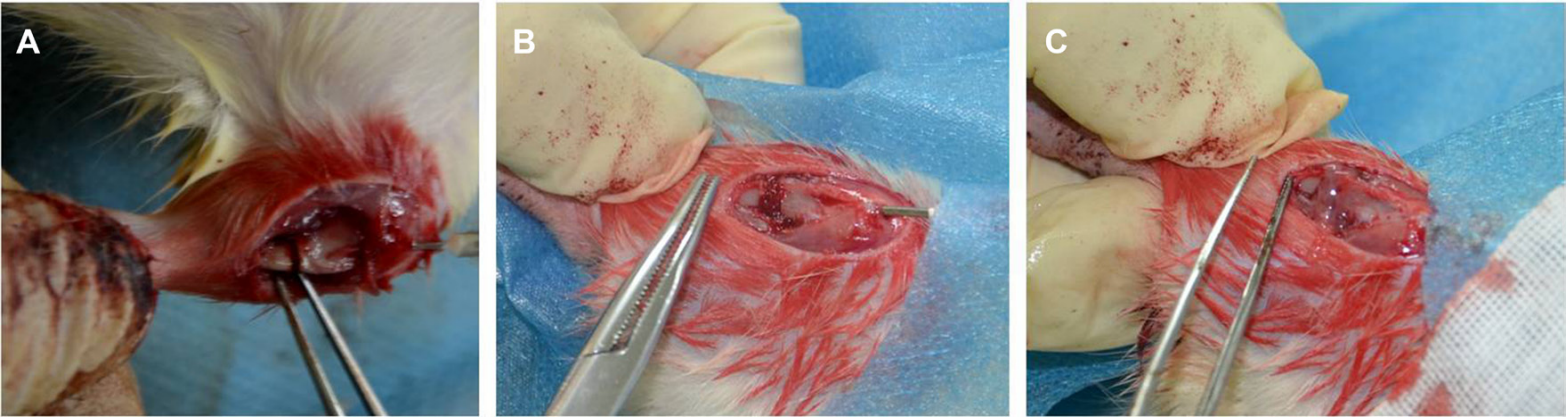
Animal model of rat tibial osteotomy (**A**) Osteotomy with needle fixation, (**B**) gelatin sponges scaffold filling the gap, and (**C**) the cell sheets wrapped the scaffold.

Repair of the bone defect in rats was examined by performing a tibial osteotomy and then monitoring the repair in response to gelatin/HMGB1-MSC sheets, gelatin/HMGB1, and gelatin (Animal model, Figure 8). Controls were left untreated. All 32 rats used in this study survived during the experimental period until their euthanization. The wounds healed well, without signs of infection, and did not limit the range of motion.

**Figure 9:**
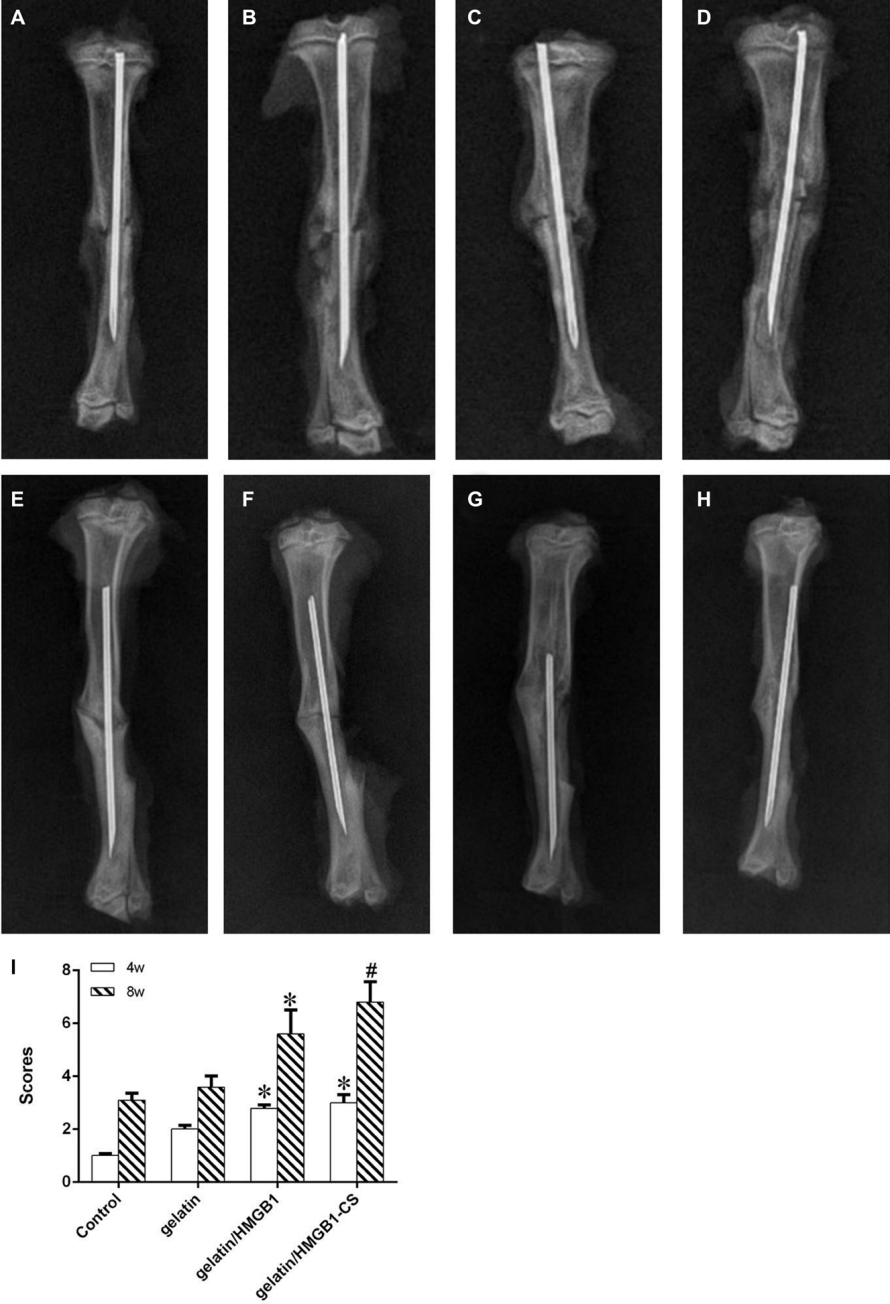
Radiographic images of osteotomized rat tibias 4 and 8 weeks postoperatively (**A**, **E**) Control group; (**B**, **F**) gelatin group; (**C**, **G**) gelatin/HMGB1 group; (**D**, **H**) gelatin/HMGB1-CS group at postoperative weeks 4 and 8, respectively. (**I**) radiographic scores of fracture sites, * *P <* 0.05 vs. the control and gelatin groups, ^#^*P <* 0.05 vs. the gelatin/HMGB1 group.

After 4 postoperative weeks, all of the tibias in the four groups showed callus formation on X-ray examination (Figure 9A–9D). Callus formation around osteotomy sites was greater in the gelatin/HMGB1-CS and gelatin/HMGB1 groups than in either the gelatin group or the control group. The cortical gap was clearly seen in the latter two groups but became obscure in the gelatin/HMGB1-CS and gelatin/HMGB1 groups. At 8 weeks postoperatively, bridging of the osseous defects was seen in the gelatin/HMGB1-CS and gelatin/HMGB1 groups (Figure 9E–9H). In the former, complete fracture union was achieved without a cortical gap; by contrast, in the gelatin group bone formation occurred but without bridging. In the untreated control group, there was minimal bone growth and the cortical gap was still apparent.

**Figure 10:**
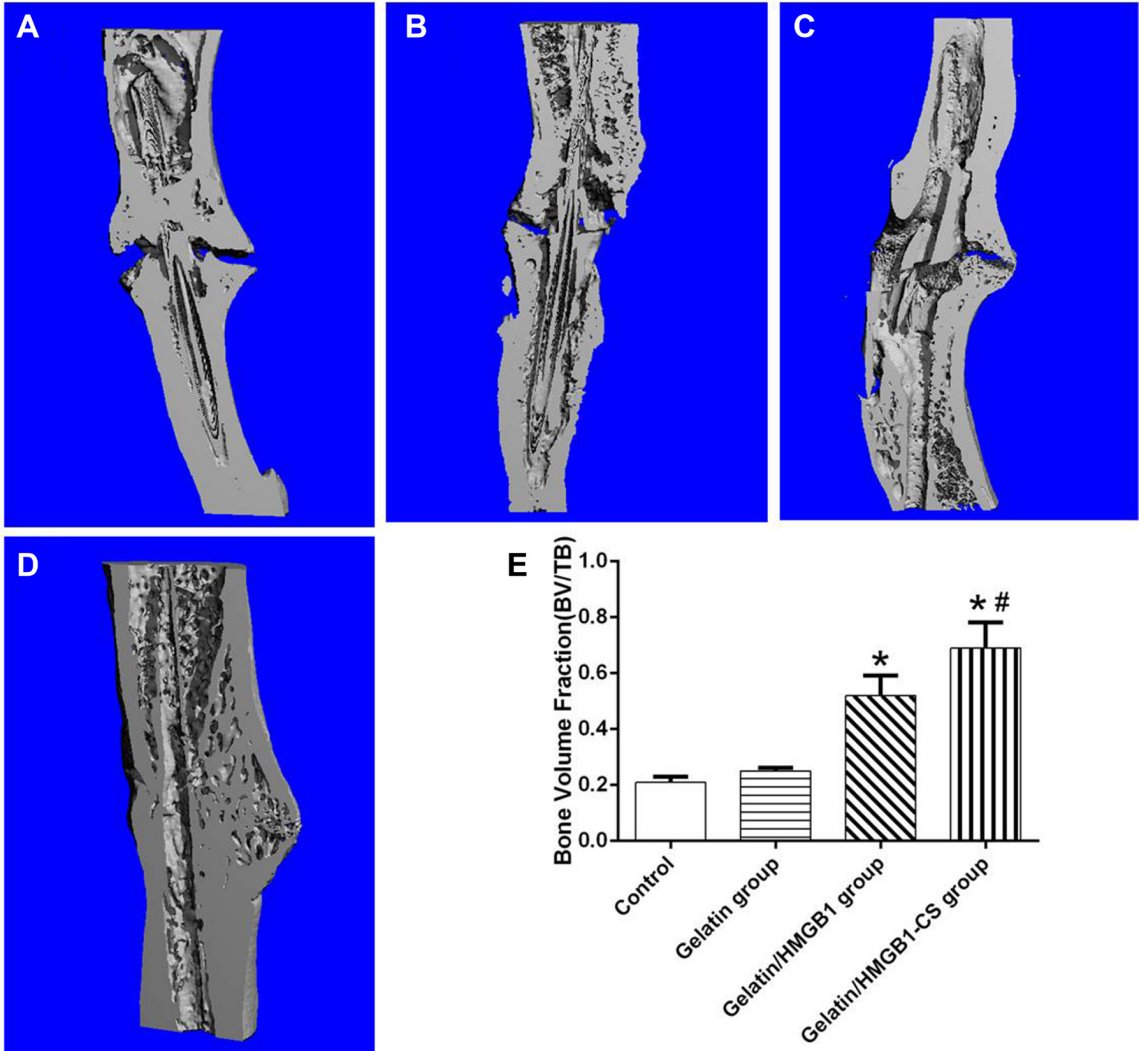
Longitudinal sections of osteotomized rat tibia 8 weeks postoperatively, as seen on microcomputed tomography (micro-CT) images (**A**) Control group; (**B**) gelatin group; (**C**) gelatin/HMGB1 group; (**D**) gelatin/HMGB1-CS group. (**E**) Micro-CT measurements of the bone volume of the corresponding areas of the same four groups versus that of the intact tibia. *P < 0.05 vs. the control or gelatin group; ^#^*P <* 0.05 vs. the gelatin/HMGB1 group.

Fracture healing among the four groups was further evaluated by micro-CT at postoperative week 8. The results showed persisting cortical gaps in the gelatin and control groups, with a wider gap in the latter (Figure 10A, 10B). In osteotomized rats treated with gelatin/HMGB1, bone formation and a partially bridged gap were evident (Figure 10C). In the gelatin/HMGB1-CS group, there was complete fracture union without a cortical gap (Figure 10D). In addition, the volume of the fractured bone was significantly higher in the gelatin/HMGB1 and gelatin/HMGB1-CS groups than in either the gelatin group or the control group. The volume of the intact contralateral tibia was significantly higher in the HMGB1-treated groups than in the other groups (Figure 10E). Moreover, the bone volume of the gelatin/HMGB1-CS treated fractured tibias was not significantly less than that of the intact contralateral tibias of the respective rats.

### Histological examination

**Figure 11:**
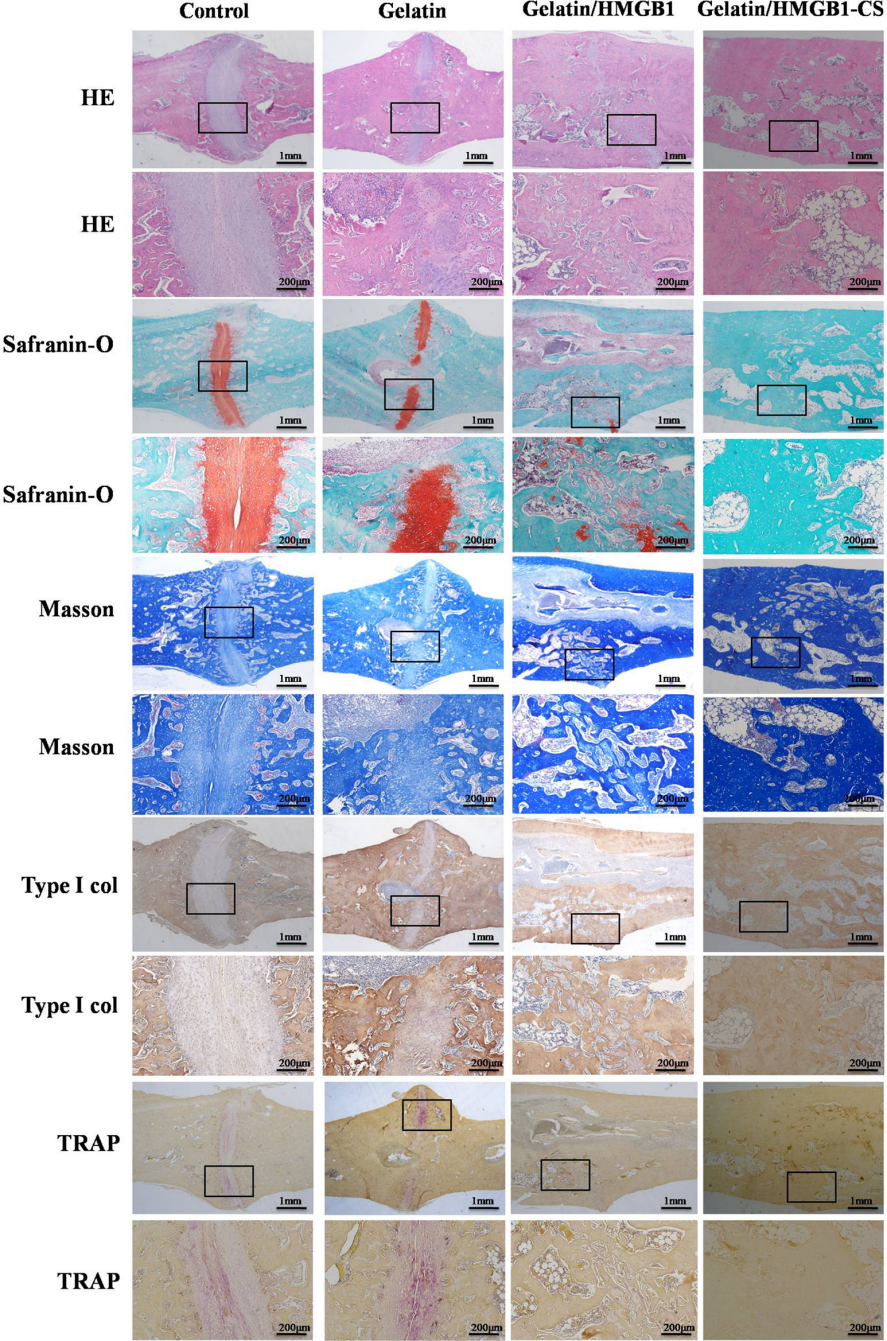
Histological and immunohistological staining of osteotomized rat tibias 8 weeks post-operatively Staining was evaluated at low (20×) and high (100×) magnification.

Callus formation was confirmed histologically in the hematoxylin and eosin-stained tibias of the four groups at 4 weeks postoperatively. Around the osteotomy sites, callus formation was greater in the gelatin/HMGB1-CS and gelatin/HMGB1 groups than in the gelatin or control groups. However, at this time point, union was not achieved in any of the fractures. Safranin-O staining showed endochondral ossification in all of the fractures. At 8 weeks postoperatively, bridging of the osseous defect was seen in the gelatin/HMGB1-CS and gelatin/HMGB1 groups (Figure 11), with complete fracture union without a cortical gap achieved in the former. In the gelatin group, bone formation occurred but without bridging of the gap, while in the control group there was very little bone growth and a clearly apparent cortical gap (Figure 11). Safranin-O and TRAP staining showed complete endochondral ossification in the gelatin/HMGB1-CS groups, whereas in the gelatin, untreated control, and gelatin/HMGB1 groups, endochondral ossification was still ongoing. Masson's trichrome staining and type I collagen immunohistochemistry revealed complete fracture repair, with collagen type I bridges through the gaps, in the gelatin/HMGB1-CS group. TRAP staining of those tibias showed fully completed bone remodeling. However, in the control and gelatin groups, the presence of numerous TRAP+ cells indicated bone remodeling by osteoclasts (Figure 11).

## DISCUSSION

To the best of our knowledge, this is the first study to explore whether HMGB1-loaded gelatin sponges served as controlled-release scaffolds accelerating fracture healing. Our results showed that the addition of HMGB1 to the gelatin sponge scaffolds improved their biocompatibility in terms of supporting MSC proliferation *in vitro*, as well as the osteogenic differentiation of MSCs and MSC sheets. We also determined that the enhanced osteogenic differentiation of MSCs was partly mediated by the STAT3 signaling pathway. In an *in vivo* study, HMGB1-gelatin sponge scaffolds combined with MSC sheets supported new bone formation in surgically treated fractures.

HMGB1 is one of the principal local stressors of the DAMP family. Extracellular HMGB1 also acts as a chemoattractant directing MSC migration and enhancing the differentiation of osteogenic and angiogenic stem cells after tissue damage [21–23]. Horst et al. [24] showed that HMGB1 expression was markedly increased in the fracture hematoma that developed in a pig model of bone fracture. In the study of Wolf et al. [25], HMGB1 increased the expression of osteoblastic marker genes in human periodontal ligament cells. In a previous study, we demonstrated that HMGB1 promoted MSC osteogenesis and migration [12, 13]. In the present study, we showed HMGB1 was effective in augmenting fracture healing when delivered locally in a gelatin sponge scaffold using a controlled-release method.

The exact molecular mechanisms by which HMGB1 accelerates fracture healing are unclear. Hsu et al. [11] found that HMGB1 promotes the osteogenic differentiation of adipose-derived stem cells and activates the ERK1/2 signal pathway, but the role of the latter in mediating the effects of HMGB1 on osteogenic differentiation was not determined. Our previous study showed that HMGB1 activates the MAPK signal pathway to promote the osteogenic differentiation of MSCs [12, 13]. In this study, the overexpression of HMGB1 in MSCs using a lentiviral vector system resulted in significant upregulation of p-STAT3 during the osteogenic induction of lenti-HMGB1 MSCs. The inhibition of STAT3 expression in MSCs during osteogenic induction, whether by siRNA knock down of STAT3 or the addition of the SH-4-54 inhibitor, prevented the osteogenic differentiation of these cells. Signaling through the JAK/STAT pathway was previously shown to be important for osteogenic differentiation [26, 27]. Disruption of the STAT3 gene in transgenic mice reduced the rate of bone formation, leading to an osteoporotic phenotype. By contrast, enhanced STAT3 activation in glycoprotein 130 knock-in mice resulted in an osteosclerotic phenotype [28]. Nicolaidou et al. [27] also found that upregulation of STAT3 in MSCs drives cellular differentiation towards osteoblasts, whereas downregulation of STAT3 by neutralizing antibodies inhibited the osteogenic differentiation of MSCs. Our results demonstrate the partial involvement of the STAT3 signaling pathway in the HMGB1-induced osteogenic induction of MSCs. STAT3 has also been shown to directly target Wnt5a, which promotes the osteogenic differentiation of MSCs [29, 30], perhaps by cross-talk between the STAT3 and Wnt signal pathways.

The establishment of a scaffold is a key element in bone tissue engineering. A suitable scaffold provides a three-dimensional structure that mimics the extracellular matrix, allowing cell attachment, migration, and proliferation [31]. In this study, we chose porous gelatin sponges as the scaffold because of their commercial availability and excellent biocompatibility. CLSM and DNA assays showed that the gelatin sponge scaffolds supported cell attachment and proliferation. In scaffolds loaded with HMGB1 to enable its controlled release, both cell attachment and proliferation increased significantly. It should be noted that, unlike clinical fractures treated with tricalcium phosphate or bioglass ceramic, which take advantage of the mechanical properties of these materials [32, 33], orthopedic surgeons prefer to use plates or intramedullary nails to achieve rigid fixation of the fracture. Thus, in patients treated by the latter approach, the mechanical properties of the scaffolds may play a minimal role in ultimately achieving repair.

The use of cell sheets has emerged as a novel technique to effectively deliver cells that promote healing while preserving cell–cell interactions and the cellular microenvironment during tissue regeneration [34]. The success achieved with cell sheets may be due to their enrichment of the extracellular matrix and their role in regulating cell differentiation [35, 36]. Using a rabbit model, we previously showed that the combination of MSC sheets with calcium sulfate-rhBMP-2 scaffolds enhanced bone regeneration [37]. Liu et al. [15] found that MSC sheets were better than MSCs in promoting osteogenic differentiation. In the rat osteotomy model used in the present study, MSC sheets combined with HMGB1-gelatin sponge scaffolds accelerated fracture healing *in vivo*. The cell sheets were wrapped around the gelatin scaffolds, thus acting as artificial periosteum [20], while the HMGB1 released from the gelatin sponge improved the microenvironment of the cell sheets and supported differentiation. In addition, the porous structure of the HMGB1-loaded gelatin sponge may induce host cell invasion and osteogenic differentiation. Our results also suggest that this simple and manageable technique can be used in the clinical setting.

Our study had several limitations. First, while this study demonstrated a role for the STAT3 signal pathway in the HMGB1-induced osteogenic differentiation of MSCs, in a previous study we showed that HMGB1 promotes MSC osteogenesis by activating the MAPK pathway [13]. Other authors reported that STAT3 promotes the osteogenic differentiation of MSCs via Wnt5a. [29, 30]. Thus, whether the HMGB1-mediated osteogenic differentiation of MSCs involves cross-talk between the STAT3 and Wnt signal pathways remains to be determined. Second, fracture repair was monitored only at 4 and 8 weeks postoperatively. To minimize the number of rats used in this study, we did not investigate the change in the quality of fracture healing at earlier and intervening time points. In *vivo* micro-CT imaging would enable us to follow the progression of fracture repair over time, as each rat would serve as its own control in these analyses. Third, long-term application of a high-density cell sheet culture induced cellular senescence. We found that cell viability within sheets declined by about 10% compared to that of MSCs at 90% confluence. However, the numbers of surviving MSCs in cell sheets were much greater than afforded by injection of cell suspensions. Cell sheets provide the extracellular matrix, which is important in tissue engineering. Fourth, loss of the stem cell characteristics of MSCs during cell sheet culture may constitute another problem. Osteogenic differentiation assay of the cell sheet showed that high-level osteogenic differentiation capacity was retained. In future, we will use ontogenetically induced cells to form cell sheets, as is often performed during myocardial or corneal tissue engineering [38, 39].

## MATERIALS AND METHODS

### Fabrication of HMGB1-releasing gelatin sponge scaffolds and characterization thereof

In order to prepare the gelatin sponge scaffolds, we cut absorbable gelatin sponges (porosity about 80%, Nanjing Jinling, China) into round discs (diameter: 5 mm and thickness: 2 mm). Next, 50 μg of recombinant human HMGB1 (Prospec) was dissolved in 500 μL phosphate-buffered saline (PBS) and uniformly pipetted onto the sponge scaffolds (10 μg HMGB1/scaffold). The HMGB1-gelatine sponge scaffolds were frozen at −80°C for 12 h then freeze-dried for 24 h.

The morphologies of the gelatin sponge scaffolds were studied by scanning electron microscopy (SEM; Hitachi, Tokyo, Japan) of samples sputter-coated with gold (Hummer; Anatech, Union City, CA, USA) in an argon atmosphere.

### *In vitro* HMGB1 release assay

The HMGB1-gelatin scaffolds were placed in 4 mL PBS and incubated without shaking at 37°C. The PBS was removed, frozen, and replenished at 1, 2, 5, 8, 10, and 12 days. PBS lacking HMGB1 served as a negative control. The HMGB1 concentrations in PBS samples were quantified by ELISA (Westang, Shanghai, China). The experiment was performed in triplicate; the data are presented as means with standard deviations (SDs).

### Bone marrow MSC isolation and differentiation assay

Eight-week-old male Sprague–Dawley (SD) rats were euthanized and their femoral bones removed using a protocol compliant with the Animal Welfare Act and approved by the Animal Care and Use Committee of Zhejiang University. The mononuclear cell fraction was isolated from each bone marrow suspension by centrifugation over Ficoll-Paque (Pharmacia, Stockholm, Sweden) at 2,500 rpm for 30 min. The cells obtained were cultured in MSC growth medium (Cyagen Biosciences, Sunnyvale, CA, USA) at 37°C in a fully humidified atmosphere under 5% (v/v) CO_2_. The medium was changed twice weekly until the cells attained confluence. The cells were then detached with trypsin, washed twice in PBS, and reseeded as described above. Cells obtained before passage 3 were used in subsequent studies.

MSC-specific marker expression was assessed by flow cytometry. Cells were washed with PBS, resuspended, and immunostained with mouse monoclonal antibodies against CD105, CD31, and CD34 (Abcam, Cambridge, UK); CD29, CD90, and CD 45 (BD Biosciences Pharmingen, San Jose, CA, USA) or corresponding isotype control (Biolegend, CA, USA). A flow cytometer (BD FACSAria SORP) was used for data acquisition.

MSC multipotency, in terms of osteogenic, chondrogenic, and adipogenic differentiation, was investigated. To induce the osteogenic lineage, cells were seeded into 12-well culture plates and cultured for 28 days in Dulbecco's modified Eagle's medium (DMEM) supplemented with 10% fetal bovine serum (FBS), 100 nM dexamethasone, and 50 mg ascorbic acid 2-phosphate/mL. Osteogenic differentiation was verified by staining with 0.5% alizarin red S (ARS) (pH 4.1) after incubation of isolated cells in 70% ethanol for 10 min. To evaluate chondrogenic differentiation, MSCs were seeded into 15-mL plastic centrifuge tubes containing DMEM with 5% FBS, 10^−7^ M dexamethasone, 50 mg l-ascorbate-2-phosphate/mL, and 10 ng transforming growth factor (TGF)-b1/mL. After centrifuging for 5 min at 550 g, the tubes were placed in a CO_2_ incubator. After 21 days of culture, the cell pellets were fixed in 10% formalin for 2 h and frozen sections (8 μm in thickness) were obtained. Chondrogenic differentiation was verified by staining with Alcian blue. Adipogenic differentiation was confirmed by inducing MSCs for 21 days in DMEM supplemented with 10% FBS, 10^−6^ M dexamethasone, 0.5 mM methyl-isobutyl-methyl-xanthine, 0.2 mM indomethacin, and 10 mg insulin/mL. The cells were fixed in 10% (v/v) formalin for 10 min and then stained with 0.18% oil red O to verify adipogenic differentiation.

### Preparation of cell sheets and MSC-gelatin constructs

To create cell sheets, 4 × 10^4^ MSCs (passage 3)/cm^2^ in MSC growth medium were seeded onto culture plates and incubated at 37°C in a humidified atmosphere under 5% CO_2_. The cells were cultured for approximately 14 days using a previously described procedure [40], rinsed twice with PBS, and then lifted as cell sheets using a scraper.

Gelatin sponge scaffolds were prepared by cutting sponges into discs (diameter 5 mm, thickness 2 mm). The MSCs were trypsinized, resuspended in MSC growth medium, seeded onto the gelatin sponge scaffolds (10^6^ cells/scaffold), and allowed to adhere to the bottoms of 12-well plates. After 1 h, the MSC gelatin constructs were cultured in MSC growth medium.

### The trypan blue exclusion assay and assay of cell sheet differentiation

The trypan blue exclusion assay was performed with the aid of a Countess automated cell counter (Invitrogen, Carlsbad, CA, USA). Briefly, 10 *μ*L amounts of cell suspension was mixed with 10 *μ*L amounts of 0.4% trypan blue and loaded into the chamber of a Countess cell counting slide. We focused the camera on the cells. Image analysis software automatically acquired data, and measured cell counts and viabilities. We also subjected cell sheets to the osteogenic, chondrogenic, and adipogenic differentiation assays described above.

### Effect of HMGB1 on MSC proliferation

MSCs were seeded in 96-well plates with a density of 10^3^ cells/well and treated with 0, 10, or 100 ng HMGB1/mL in MSC growth medium for 1, 3, and 7 days. Then we performed CCK-8 (cell counting kit ; Dojindo, Kumamoto, Japan) test to measure cell proliferation following the manufacturer's instructions. The medium was replaced with 100 μL of DMEM and 10 μL of CCK-8 at each time point. Followed by incubation at 37°C for 4 hours, the absorbance at 450 nm was measured by a microplate reader (Bio-Rad, USA).

### Effect of gelatin-HMGB1 scaffolds on MSC proliferation

The numbers of cells in scaffolds were measured by the amounts of cellular DNA using the Hoechst 33258 dye (Sigma, USA) on days 0, 2, 4, 6, and 8. Briefly, cell-scaffold constructs were crushed and incubated with proteinase K (Sigma) at 45°C overnight. Lysates (500 μL) were then mixed with 500 μL amounts of Hoechst 33258 solution (1 μg/mL; Sigma) and the fluorescence intensities at 450 nm were measured spectrophotometrically (SpectraMax M5; Molecular Devices, Sunnyvale, CA, USA). DNA contents were determined using a calibration curve prepared employing calf thymus DNA (Sigma) as the standard.

### MSC distributions in gelatin and gelatin-HMGB1 scaffolds

MSC distributions in scaffolds were studied by confocal laser scanning microscopy (CLSM) (TCS SP8; Leica, Wetzlar, Germany). Cells were labeled with the fluorescent dye CM-DiI (Molecular Probes, Eugene, OR, USA) according to the manufacturer's protocol. Briefly, 50 μL of a sterile stock solution (1 mg/mL) of the dye was added to a suspension of 1 × 10^6^ cells, resulting in a final CM-DiI concentration of 4 μg/mL. After 5 min incubation at room temperature, and 15 min incubation at 4°C, the cells were rinsed at least three times with PBS to remove all unbound CM-DiI. The MSC-gelatin constructs were prepared as described in Section 2.4. After 7 days, the scaffolds were removed from the culture plate, gently rinsed with PBS, and sagittal sections (50 μm in thickness) were obtained and frozen. The sections were examined by CLSM; z-stack images (20 confocal images; 2-μm steps) were acquired. Three-dimensional reconstructions were created using a 10× objective and Elements software (Leica).

### Alizarin red staining

Mineral deposition was assessed by ARS (Cyagen Biosciences, Guangzhou, China). MSCs were fixed in 4% paraformaldehyde (Sangon Biotech, Shanghai, China) for 10 min at room temperature, washed with distilled water, treated with ARS (0.5%) for 30 min at room temperature, and rinsed with distilled water. The absorbances at 560 nm of 200-μL suspensions of stained cells in 96-well plates were measured using a microplate reader (ELX808; BioTek, Winooski, VT, USA). The readings were normalized to the total protein concentrations.

### Real-time quantitative polymerase chain reaction (RT-qPCR)

MSCs were seeded into six-well plates (4 × 10^4^ cells/well) containing osteogenic induction medium (OIM; DMEM, 10% FBS, 100 IU penicillin/streptomycin/mL, 100 nM dexamethasone, 0.2 mM ascorbic acid, and 10 mM β-glycerophosphate) and 100 ng HMGB1/mL. Total RNAs were isolated from cells cultured for 3 and 7 days, using the RNAiso reagent (TaKaRa Bio Inc., Shiga, Japan). Reverse transcription was performed using 2 μg amounts of total RNA, Prime ScriptRT kit reagents, and gDNA Eraser (TaKaRa). The levels of mRNAs encoding alkaline phosphatase (ALP), osteocalcin (OCN), runt-related transcription factor 2 (RUNX2), and collagen α1 type I (COL1A1) were determined using a StepOnePlus real-time PCR system (Applied Biosystems Inc., Carlsbad, CA, USA) and the SYBR Premix Ex Taq (TaKaRa) under the following conditions: 95°C for 30 s followed by 40 cycles of 95°C for 5 s and 60°C for 30 s. GAPDH served as an internal control, allowing differences among samples to be adjusted for. DNA concentrations were calculated using the 2^−∆ ∆ Ct^ method (Livak & Schmittgen, 2001). All primers used in this experiment were synthesized by Sangon Biotech and are listed in Table 1.

**Table 1:** Primer sequences for real-time quantitative polymerase chain reaction

Genes	Forward primer	Reverse primer
ALP	GACACGCTGAGCCTCGTCACT	CCTGGACCGTTTCCGTATAGG
RUNX2	CAAGTGGCCAGGTTCAACGA	TGTGAAGACCGTTATGGTCAAAGTG
COL1A1	CCTGCTGGCAAGAGTGGTGAT	CAAGTTCCGGTGTGACTCGTG
OCN	AGGACCCTCTCTCTGCTCAC	GCTCACACACCTCCCT
GAPDH	CTCAGTTGCTGAGGAGTCCC	ATTCGAGAGAAGGGAGGGCT

### Lentiviral packaging and cell infection

The HMGB1-overexpression lentiviral vector (lenti-HMGB1) and lenti-green fluorescent protein [GFP] (the negative control) were purchased from Cyagen Biosciences. Transfections were performed according to the manufacturer's instructions. Briefly, MSCs were incubated with lentiviral particles and 5 μg polybrene/mL in growth medium. After 12 h, the infection medium was replaced with MSC medium. The cells were passaged on day 3 for use in further experiments. HMGB1 expression was quantified by RT-PCR and Western blotting.

### siRNA transfection

STAT3 depletion was achieved by transfecting the MSCs with an siRNA (GenePharma, Shanghai, China) that had the following sequence: sense 5′-GCAGGAU CUAGAACAGAAATT-3′ and antisense 5′-UUUCUGUU CUAGAUCCUGCTT-3′. The cells were seeded into six-well plates (3 × 10^5^ cells/well) and transfected with the siRNA duplex for 24 h using Lipofectamine 2000 (Invitrogen, Ontario, Canada) according to the manufacturer's instructions. They were harvested at 72 h prior to RNA and protein extraction

### Immunofluorescence

Cells were cultured in induction medium in 12-well plates. COL1A1, RUNX2,and p-STAT3 were detected by fluorescence microscopy (Leica, Germany). Briefly, after 10 min of fixation at room temperature in 4% paraformaldehyde, cells were blocked for 30 min in 0.04% Triton X-100 and 5% bovine serum albumin and then incubated overnight with anti-COL1A1 (1:500; Abcam), anti RUNX2 (1:1,600; Cell Signaling Technology, Shanghai, China), or anti-p-STAT3 (1:100; Cell Signaling Technology). The cells were then incubated with a secondary antibody conjugated to a fluorescent material (Beyotime, Haimen, China) for 120 min. The cell nuclei were stained with DAPI (KeyGen Biotech, Nanjing, China) for 4 min. Immunofluorescence was observed under a fluorescence microscope (Leica, Germany).

### Western blotting

Cells were lysed in RIPA lysis buffer (Beyotime, China) and subjected to SDS-PAGE on 10% polyacrylamide gels. The proteins were blotted onto PVDF membranes (Millipore, Shanghai, China). After blocking in 5% non-fat milk for 2 h, the membranes were incubated overnight at 4°C with antibodies specific for GAPDH (1:1,500; Cell Signaling Technology), RUNX2 (1:1,600; Cell Signaling Technology), p-STAT3 (1:1,000; Cell Signaling Technology), or HMGB1 (1:7,000; Abcam); and were then incubated with horseradish-peroxidase-conjugated goat anti-rabbit IgG (1:1,500; Cell Signaling Technology) (the secondary antibody) for 2 h at room temperature. Immunoreactive bands were detected using an enhanced chemiluminescence detection reagent (Millipore) and further visualized by exposing the blots to X-ray film (Bio-Rad) for 0.1–2 min. Protein expression was quantified by measuring the ratio of the absorbances of proteins of interest to that of the internal control (GAPDH).

### Animals and surgery

All animal experiments were performed under the guidelines of the Animal Care and Use Committee of Zhejiang University and the experimental designs were approved by that Committee. Thirty-two 12–13-week-old male SD rats (about 280–320 g) were supplied by the Animal Center of Zhejiang Academy of Medical Sciences and were housed in a light- and temperature-controlled environment.

The 32 animals underwent 64 osteotomy procedures and were randomly divided into four groups. The control group was left untreated (*n* = 16 tibiae). The gelatin group was treated with gelatin sponges alone (*n* = 16 tibiae). In gelatin/HMGB1 group, rats received gelatin sponge/HMGB1 control-releasing composites (*n* = 16 tibiae). The gelatin/HMGB1-CS group was treated with a gelatin sponge/HMGB1 controlled-release composite combined with cell sheets (*n* = 16 tibiae). Histological evaluations were performed 4 and 8 weeks postoperatively.

All rats were anesthetized with 0.3% pentobarbital sodium (Sigma, USA) given intraperitoneally at ~2.3 mL/kg body weight (30 mg/kg body weight). A lateral incision was made on the proximal tibia and the muscle was longitudinally divided to expose the tibia. The periosteum was removed from the osteotomy sites of the tibia. A mini-drill was used to perform a transverse osteotomy (from front to back) in the proximal one-third of the tibia, creating a 1 mm gap. We then enlarged the gap with a forceps and placed the gelatin-sponge scaffold into the gap. All rats were treated as described above. A small hole was drilled in the tibial tubercle to allow insertion of a 21-gauge needle that extended to the distal end of the tibia, affording loose fixation. We then trimmed excess scaffold from around the osteotomy site and wrapped stem cell sheets around the site (Figure 8).

### Radiographic examination

All animals were euthanized at 4 and 8 weeks postoperatively by intraperitoneal injection of an overdose of pentobarbital sodium. Radiographic examination was performed with a Mo-targeting mammographic device (22 kV, 250 mAS; General Electric, USA). Fracture healing was evaluated in greater detail using 3D reconstructions obtained by microcomputed tomography (micro-CT) (mCT 40; Scanco Medical, Bassersdorf, Switzerland) and images obtained using a 10-mm focal spot microfocus X-ray tube. The scout view X-ray images were used for radiographic evaluation. For quantitation, we used a scoring scale based on cortical rebridgement and healing acceleration (Table 2). All evaluations were triple-blinded. The region of the bone defect was scanned using an isotropic voxel size of 20 mm, at 70 keV of energy, with a 400-ms integration time. We collected 420 images of each sample. The inner 3 mm of the osteotomy area was examined to ensure that only newly formed bone was included in analyses. All images were thresholded using an adaptive-iterative algorithm [41, 42] and morphometric variables were computed from binarized images [43]. The bone volume fraction (BV/TV), including the area of bone formation, was calculated by standard 3D microstructural analysis.

**Table 2:** X-ray scoring scale of fracture healing

Scores	X-ray of fracture sites
0	No bridging, no callus formation
1	No bridging, initiation of a small amount callus
2	No bridging, obvious initial callus formation near fracture
3	No bridging, marked callus formation near and around fracture site
4	Rebridging of at least one of the cortices, marked callus formation near and around fracture site
5	Rebridging of at least one of the cortices, marked and complete callus formation around fracture site
6	Rebridging of both cortices, and/or some resolution of the callus
7	Clear rebridging of both cortices and resolution of the callus

### Histological staining and TRAP

Specimens were fixed in 10% neutral formalin, decalcified in 10% EDTA (pH 7.4), sectioned longitudinally through the medullary canal, and embedded in paraffin. Sagittal sections (4 μm in thickness) were obtained from the center of the defect and stained with hematoxylin, safranin-O, and Masson's trichrome stain. Tartrate-resistant acid phosphatase (TRAP) expression was evaluated using an acid phosphatase staining kit (Jiancheng, Tianjin, China) with 50 mM tartrate as the substrate.

### Type I collagen immunohistochemistry

Type I collagen expression was detected using a rabbit anti-rat type I collagen monoclonal antibody (1:100 in PBS; Abcam) employing the avidin-biotin immunoperoxidase method. All histological sections were observed under an Olympus BX-51 light microscope (Olympus, Tokyo, Japan).

### Statistical analysis

Statistical analysis was performed with the aid of SPSS software (ver. 17.0; SPSS Inc., Chicago, IL, USA). Statistical significance was evaluated using the non-parametric Mann–Whitney and Wilcoxon signed-rank tests. All data are presented as means ± SDs. A *p value* ≤ 0.05 was considered to indicate statistical significance.

## CONCLUSIONS

In conclusion, this study showed that HMGB1-loaded gelatin sponges, as controlled-release scaffolds, combined with MSC sheets enhance fracture healing. HMGB1 promoted fracture healing partly via the STAT3 signaling pathway. Commercially available natural gelatin sponges may be used as carriers accelerating fracture healing.

## SUPPLEMENTARY MATERIALS FIGURES



## Abbreviations

MSC: mesenchymal stem cells
CS: cell sheets
HMGB1: high mobility group box-1 protein
BMP-2: bone morphogenetic protein-2
α-MEM: minimal essential medium α
FBS: fetal bovine serum
DMSO: dimethyl sulfoxide
OIM: osteogenic induction medium
ARS: alizarin red S
