# Supplementary material for: Local delivery of HMGB1 in gelatin sponge scaffolds combined with mesenchymal stem cell sheets to accelerate fracture healing
Source: Oncotarget. 2017 Apr 6;8(26):42098–115. doi: 10.18632/oncotarget.16887 (PMC5522052; doi:10.18632/oncotarget.16887)
Supplement: Supplementary file 1 [file oncotarget-08-42098-s001.pdf]

Local delivery of HMGB1 in gelatin sponge scaffolds combined with mesenchymal stem cell sheets to accelerate fracture healing

Supplementary Materials

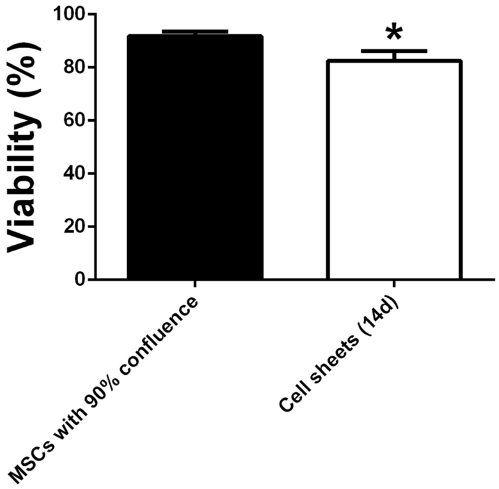

Supplementary Figure 1: The cell viability of the cell sheets and MSCs with 90% confluence.

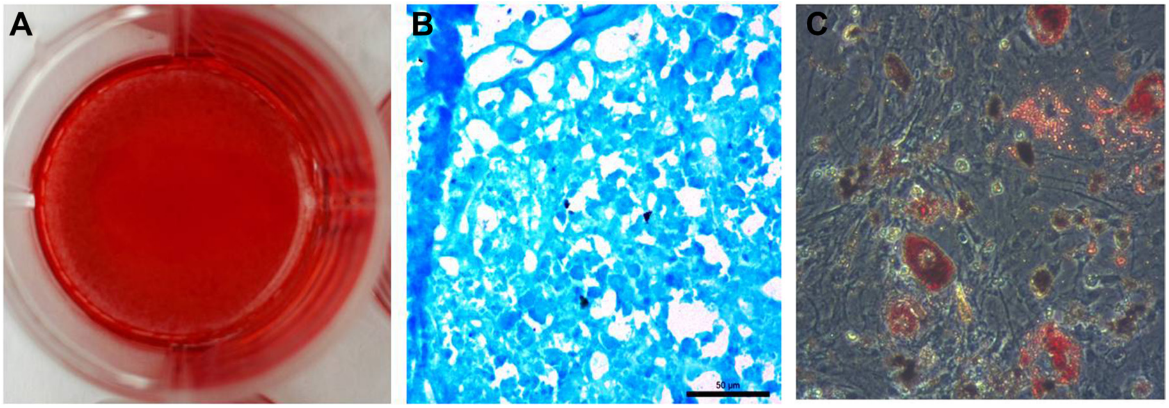

Supplementary Figure 2: (**A**) Alizarin red, (**B**) Alcian blue, and (**C**) oil red O staining of the cell sheet.
